# Perceptions on the right to adequate food after a major landslide disaster: a cross-sectional survey of two districts in Uganda

**DOI:** 10.1186/s12914-015-0047-x

**Published:** 2015-04-25

**Authors:** Peter M Rukundo, Per O Iversen, Bård A Andreassen, Arne Oshaug, Joyce Kikafunda, Byaruhanga Rukooko

**Affiliations:** Department of Human Nutrition and Home Economics, Kyambogo University, Kampala, Uganda; Department of Nutrition, Institute of Basic Medical Sciences, University of Oslo, Oslo, Norway; Department of Haematology, Oslo University Hospital, Oslo, Norway; Norwegian Centre for Human Rights, Faculty of Law, University of Oslo, Oslo, Norway; Faculty of Applied Health Sciences, Oslo and Akershus, University College of Applied Sciences, Oslo, Norway; School of Food Technology, Nutrition and Bio-engineering, Makerere University, Kampala, Uganda; School of Liberal and Performing Arts, Makerere University, Kampala, Uganda

**Keywords:** Rights holders’, Perceptions, Right to adequate food, Landslide, Uganda

## Abstract

**Background:**

Despite the instruments on the right to adequate food adopted by the United Nations, there exists limited information on how this right is perceived. Following a major 2010 landslide disaster in the Bududa district of Eastern Uganda and the resettlement of some affected households into the Kiryandongo district in Western Uganda, we surveyed both districts to explore perceptions about the right to adequate food among households with different experiences; disaster-affected and controls.

**Methods:**

We deployed qualitative and quantitative techniques to a cross-sectional survey. The index respondent was the head of each randomly selected household from the landslide affected communities and controls from a bordering sub-county. Data was collected by interviews and focus group discussions (FGDs). Structured entries were tested statistically to report associations using Pearson’s Chi-square at the 95% CI. Information from FGDs was transcribed, coded, sequenced and patterned. Findings from both techniques were triangulated to facilitate interpretations.

**Results:**

Analysis included 1,078 interview entries and 12 FGDs. Significant differences between the affected and control households (P < 0.05) were observed with: age; education level; religious affiliation; existence of assets that complement food source; and having received relief food. Analysis between groups showed differences in responses on: whether everyone has a right to adequate food; who was supposed to supply relief food; whether relief food was adequate; and preferred choice on the means to ensure the right to adequate food. FGDs emphasized that access to land was the most important means to food and income. Affected households desired remedial interventions especially alternative land for livelihood. Despite the provision of adequate relief food being a state’s obligation, there was no opportunity to exercise choice and preference. Comprehension and awareness of accountability and transparency issues was also low.

**Conclusion:**

Though a significant proportion of participants affirmed they have a right to adequate food, relief food was largely perceived as insufficient. Given the high regard for land as a preferred remedy, a resettlement policy is of the essence to streamline post-landslide displacement and resettlement. Information materials need to be assembled and disseminated to stimulate awareness and debate on the right to adequate food.

## Background

The right to adequate food has been established in international human rights declarations and treaties for over 65 years [1,2]. In 1999, the United Nations clarified in its General Comment 12 (GC 12) that this right is realized when every man, woman and child, alone or in the community with others, has physical and economic access at all times to adequate food or means for its procurement [3]. Subsequently, this landmark achievement of GC 12 was expounded by an inter-governmental working group that yielded the Voluntary Guidelines (VGs) to support the progressive realization of the right to adequate food in the context of national food security; a tool of nineteen guidelines that was adopted by the Council of the Food and Agriculture Organization of the United Nations in 2004 [4].

At the country level, it is expected that the relevant human rights tools are domesticated and translated into law. However, given that studies investigating perceptions on the right to adequate food in the context of landslide disasters are lacking, it may be unlikely to assume that rights holders’ have perceived their right to adequate food and related state obligations in the same form and substance specified by the GC 12 and VGs. As observed by Eide and colleagues [5], perceptions about the right to adequate food may depend on whether or not an individual is facing an eminent threat of starvation; households suffering the effects of hunger may perceive the right to adequate food as a real matter of urgency, while some other sections of society who are not faced by such problems may often not perceive it with urgency. Indeed hunger and under-nutrition are serious forms of deprivation which severely diminish human dignity and quality of life, and may lead to death. Estimates on this global problem show that the proportion of the people living on the margins of hunger are still unacceptably high [6]. At the end of 2013, more than one in eight people in the world were suffering from the consequences of chronic hunger and not having enough food to eat [7,8].

In Uganda there are relevant measures to support the right to adequate food. Its Constitution adopted in 1995 recognised the right to adequate food and other economic, social and cultural rights, and has committed as a matter of directive principle of state policy to ensure food security and nutrition for all [9]. A Food and Nutrition Policy that recognises the right to adequate food was also adopted with a leading principle stipulating that a rights-based approach would be adopted in the implementation of food and nutrition programmes [10]. However, poor nutrition is still a concern as recent estimates have shown an increase in one million undernourished people; from about 9 million in 2010 [7,11] to about 10 million people in 2014, representing an increase from 25% to 28% of the total population [8].

Implementation of a rights-based approach to food and nutrition is also facing perception challenges. A study targeting duty bearers in Uganda revealed that some of them perceived this right with ambiguity and contempt due to low awareness and appreciation of human rights [12]. In another case, relevant duty bearers were found to have low knowledge and awareness on the right to adequate food and related state obligations [13]. Moreover, there is information gap on how vulnerable rights holders perceive their right to adequate food, especially those affected by disasters. Such gaps have potential to slow public debate and actions of rights holders in their demand for state actions against hunger and undernutrition [14].

The main objective of this study was to explore the perceptions about the right to adequate food in the aftermath of a landslide disaster that occurred in March 2010 in the Bududa district of Eastern Uganda. The rationale was based on existing evidence that this was the worst natural disaster event in the country’s history: an estimated 350 persons are reported to have died [15,16], while about 10% of the 10,000 affected people were resettled over 300 kilometres away in the Mutunda sub-county of the Kiryandongo district in Western Uganda [17]. We surveyed the two districts to compare disaster affected households and counterparts who were not affected by the disaster (controls). In effect, we sought to establish whether in the aftermath of the major 2010 landslide disaster, people perceived of their right to adequate food differently when they are victims of a disaster and subsequent experiences, than those who did not experience the same events. Relief food which constitutes a major component of the state’s obligation to fulfil, including by way of facilitation and provision, of the right to adequate food [3,18], was a point of departure for the study.

## Methods

### Design and setting

This study is part of a trial analysing the disaster preparedness and emergency response system for ensuring the right to adequate food in Uganda. We used both quantitative and qualitative techniques in a cross-sectional, multi-stage survey involving interviews and focus group discussions with rights holders’ in two purposively selected districts in Uganda in the period between August 2012 and May 2013. The Bududa district was chosen because it is disaster-prone and its sub-county called Bukalasi was the site of the devastating landslide in 2010. In addition, the Kiryandongo district was selected because it was host to about 10% of the disaster-affected households who accepted to be voluntarily resettled by the Government into the Mutunda sub-county of the district.

In each district, the study participants were categorized as either affected or controls. The affected group comprised landslide disaster-affected households while the control group comprised households from one randomly selected sub-county bordering the sub-county with the disaster-affected group. In the Bududa district, the affected group were selected from the Bukalasi sub-county, where several households, an entire trading centre and a health facility were buried by the disaster [15,17]. The control households were therefore selected from the Bubiita sub-county, one of the neighbouring sub-counties of Bukalasi. In the Kiryandongo district, the affected households were selected from the resettled landslide disaster victims in the Mutunda sub-county, while the controls were selected from the Kiryandongo sub-county, a neighbour of the Mutunda sub-county that shares the same name of the district.

The two districts were examined independently given that they present different experiences and unique socio-cultural, geographical and ecological attributes. The Bududa district is of a hilly terrain given its location on the foot of the South-Western slopes of the Mount Elgon Volcano in Eastern Uganda [15]. Average precipitation of the area is above 1,500 millimetres (mm) of rainfall per year, supporting bi-annual seasons for crop planting and harvesting [15,19]. Furthermore, the population is mainly Lumusaba speaking and the national housing and population census of 2002 enumerated over 123,000 people and a population growth rate of 3.8% [20]. However, estimates for 2010 and 2011 were projected at over 167,000 and 173,000 people in Bududa district [21]. On the other hand, Kiryandongo district is of a flat terrain and located in Western Uganda, approximately 250 kilometres northwest of Kampala city. The rainfall is bimodal with an average of 1,200 mm [22]. Whereas the estimates from the national housing and population census of 2002 reported that Kiryandongo had a population of about 190,000 people [20], the population of this district has fluctuated over time mainly due to its suitability as a resettlement area for refugees and internally displaced persons [23-25]. As such, data from the two districts was not pooled.

### Quantitative techniques

#### Sample size computation

Given the absence of reliable sample size estimators to study perceptions about food as a human right in the context of landslide disaster, we adopted relevant estimators for surveying food insecurity and diet related variables. We assumed that the 19% national prevalence of undernourishment reported in the Uganda Nutrition Action Plan 2011-2016 [11] was relevant for the control groups, and 29% for the affected groups. Using an equal ratio of control to affected group at the 95% confidence level and 80% power, we computed a cross-sectional sample of 576 households per district: 288 per group. An extra 12 households was added to each group in each district to compensate for possible non-response [21,26]. We therefore targeted 300 households per group and a total of 600 households per district; hence a total of 1,200 households were eligible for inclusion into the study from the two districts combined.

#### Selection of households

Due to the community organization and the geographical set-up of the study areas, we adopted a multi-stage sampling procedure involving three stages to select the households for quantitative interviews. This commenced with the selection, by simple ballot, of the control sub-county from a list of sub-counties neighbouring the sub-county with affected households. The assumption was that the households’ conditions were relatively similar prior to the disaster events of 2010. At the second stage, all the villages in each of the designated affected and control sub-counties were listed and households assigned into 25 villages using the probability proportion to size technique, hence a total of 50 villages per district, i.e. 100 villages in both districts combined. The third stage involved selecting 12 representative households in each village from the household lists that were generated with the assistance of the village authorities during a mapping and listing exercise. Using computer-generated simple randomisation, random numbers were obtained from a range of an ascending numbered list of village households. Household whose position on the numbered list matched with the random numbers were identified for interviews.

#### Recruitment and training of assistants

Using a competitive process whose minimum requirements were 15 years of schooling and evidence of a college diploma, we recruited one overall fieldwork assistant for the two districts and 10 data collection assistants per district. Fluency in the local languages of Lumusaba in Bududa district and Runyakitara in Kiryandongo district were also a key pre-requisite for selecting assistants. The assistants were trained on the questionnaire content and probing skills before the survey tools were pre-tested and adapted.

#### The questionnaire and the interviews

The questionnaire was a pre-coded and structured tool with mainly closed ended questions on socio-demographic characteristics and perceptions on the right to adequate food during disaster response in Uganda. Nine questions about socio-demographic variables targeted to capture information from the head of the household with regard to: gender; age; household size; education level; affiliation to common religious denominations; main source of livelihood; existence of assets to complement food source; and whether the household had received any form of relief food in the three years preceding the interview. In addition, seven questions on awareness and perceptions about the right to adequate food and disaster relief food were asked: whether everyone has a right to adequate food; whether the 2010 landslide disaster in the Bududa district affected access to adequate food; whether the emergency response to the 2010 disaster was considered satisfactory; who is supposed to supply relief food in Uganda; whether relief food supplied to Ugandans is of good quality; whether relief food supplied to Ugandans is of sufficient quantity; and the preferred means to ensure the right to adequate food of disaster victims.

Potential information bias was minimized by translation of terms from the local language back into English, pre-testing of the questionnaire prior to data collection, and flexibility in conducting interviews in a local language in cases where the interviewee could not communicate fluently in English. In addition, a household mapping and listing exercise was done prior to household randomization to overcome sampling bias. We also stratified socio-demographic variables into sub-groups so to minimize confounding bias during the analysis.

Interviews were conducted with the male or female head of the household. Pre-tested households were excluded from the final randomisation. Although we preferred to interview women respondents due to their role in food and nutrition security, the head of the household who was available and willing to participate was the one interviewed.

#### Statistical analysis

We used version 21 of IBM SPSS to report associations between categorical variables using the Pearson *x*^2^ test at the P < 0.05 level of significance. Given the two different districts, and the different experiences faced by disaster affected and control households therein, analysis between groups was independent for each district. Moreover, given the ecological nature of the disaster, the controls could not be selected from within the same area of the affected; hence we had two heterogeneous groups that limited pooled analysis.

### Qualitative techniques

#### Constitution of focus groups

Given the suitability of focus group discussions (FGD) in exploring experiences and group dynamics [27,28], we held separate discussions with three groups of people in each of the affected and control groups in the two districts: youth aged 18 to 35 years; adult men above 35 years of age; and women in the same age range as adult men. A total of 12 FGD were held. Discussants were sampled independently from households who were not randomised for quantitative interviews. The leadership in each sub-county assisted to mobilise the FGD participants.

We targeted 6-8 participants for each FGD. Participants were told beforehand to be at liberty to discuss in English or their native languages, and that all answers were equally important. An interpreter who was fluent in both English and the local languages facilitated the immediate translation of what was being said in the local language. Both the audio- and written records were collected with permission of the participants. In the Bududa district, all the FGD meetings were convened at the headquarters of the Bukalasi sub-county and the Bubiita sub-county for the affected and controls respectively. In the Kiryandongo districts, FGD for the affected communities were held at a health facility in the Mutunda sub-county. Discussions with the controls were held at the Kiryandongo sub-county head-quarters.

#### Focus group questions

The three key words food, diet and human rights were used as leads for easy comprehension of two leading questions that were meant to progressively explore the right to adequate food content. The two main FGD questions were: how was the food and diet of the people of Bududa district affected following the landslide disaster of 2010; and, how were human rights issues of participation, accountability, non-discrimination and transparency taken into consideration during the response of public authorities to the disaster?

Relevant phrases about the right to adequate food were deployed to probe and gain more insights in the context of the landslide disaster. With regard to the first FGD question on the right to adequate food, some of the phrases of the question posed included: *‘the food situation in the area’; ‘sufficient food in the homes’; ‘varieties of food’; ‘food production’; ‘affording the price of food in the market’; ‘type and quality of relief food’; ‘choice and preference of relief food’;* and *‘food that is forbidden or accepted by culture’*. Although these might not have been exhaustive about the right to adequate food, they provided a basis for further discussions.

For the second question regarding the components of a rights based approach, the main phrases used to solicit responses to the question included: *‘whether people were participating in decisions about relief food’; ‘whether people understand how relief was being managed’; ‘whether people had access to information on how relief food was being mobilised and managed’*; and *‘whether they knew about the budget and resources involved when disaster relief food was distributed by the government’*. A few choices of phrases were selected to probe this question given the complexity of interpreting a rights-based approach in such a rural setting.

#### Analysis of focus group data

A de-brief was held after each FGD to identify and highlight the key messages for further scrutiny. On transcription, messages were coded under similar themes, sequenced and patterned to obtain relevant narratives and anecdotes.

### Ethical approval

The study was approved by the Uganda National Council of Science and Technology; reference number SS 2885. A sensitization meeting was held with district authorities’ to inform them about the main aspects of the study. Confidentiality, informed consent and all other standards set by the Helsinki declaration were upheld.

## Results

### Socio-demographic features in the study population

**Figure 1:**
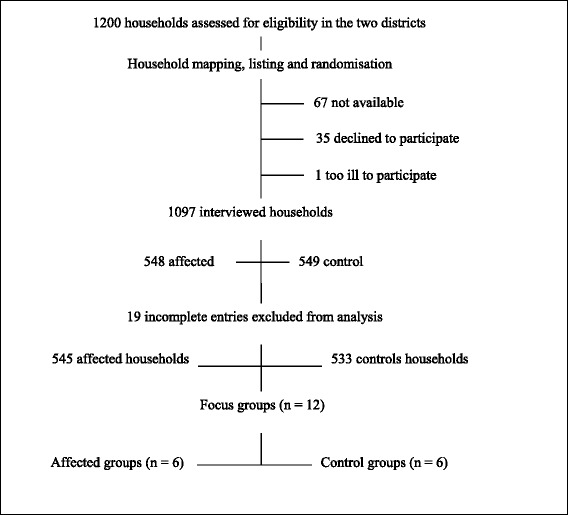
Inclusion into the quantitative survey and focus group discussions.

As shown in Figure 1, a total of 1,078 household entries were analysed out of the 1,097 representatives that were interviewed as part of the quantitative survey. On the other hand, a total of 79 discussants participated in the 12 focus group sessions that were held. The average number of participants in each focus group was seven (range 6-10) excluding the facilitator, interpreter and recording assistant. Men came in more numbers (n = 29) followed by women (n = 26) and the youth (n = 24). The duration of a session was in the range of 60-90 minutes.

Socio-demographic characteristics shown in Table 1 indicate that the control households in the Bududa district were older with a mean (SD) of 43.6 (16.0) years compared to 38.9 (17.0) years in their affected group counterparts (P < 0.01). In the Kiryandongo district the affected households were older with an average of 40.0 (11.9) years compared to 37.6 (14.0) years in their control counterparts (P = 0.04). Household size was larger in the control households in the Bududa district, with an average of more than six members compared to five members in their counterparts in the affected group (P < 0.01). In the Kiryandongo district, there was no significant difference in household size between the affected and control groups.

**Table 1:** **Socio-demographic characteristics of interviewed heads of the households**

**Variable**	**Bududa district (n = 555)**			**P values**	**Kiryandongo district (n = 523)**			**P values**
	**Affected (n = 285)**	**Controls (n = 270)**	**Total (% or SD)**		**Affected (n = 260)**	**Controls (n = 263)**	**Total (% or SD)**	
*Gender of the interviewed head of the household*								
Male	189	184	373 (67.2)	0.65	125	148	273 (52.2)	0.07
Female	96	86	182 (32.8)		135	115	250 (47.8)	
*Age of the interviewed head of the household*								
Mean (SD)	38.9 (17.0)	43.6 (16.0)	41.2 (16.7)	<0.01	40.0 (11.9)	37.6 (14.0)	38.8 (13.1)	0.04
*Household size*								
Mean (SD)	5.0 (3.2)	6.4 (3.0)	5.7 (3.2)	<0.01	6.4 (2.7)	6.1 (2.8)	6.3 (2.8)	0.14
*Education level of the head of the household*								
None	50	78	128 (23.1)	<0.01	26	55	81 (15.5)	<0.01
Primary	213	140	353 (63.6)		193	178	371 (70.9)	
Secondary	19	46	65 (11.7)		39	25	64 (12.2)	
≥ College	3	6	9 (1.6)		2	5	7 (1.3)	
*Religious faith affiliation*								
Anglican	137	153	290 (52.3)	<0.01	103	78	181 (34.6)	0.01
Catholic	83	80	163 (29.4)		77	83	160 (30.6)	
Islam	12	10	22 (4.0)		24	25	49 (9.4)	
Pentecostal and evangelical	46	20	66 (11.9)		19	45	64 (12.2)	
None and others	7	7	14 (2.5)		37	32	69 (13.2)	
*Main source of livelihood*								
Farming	271	229	500 (90.1)	<0.01	225	223	448 (85.7)	0.29
Wage	1	10	11 (2.0)		6	13	19 (3.6)	
Trader	6	22	28 (5.1)		26	19	45 (8.6)	
Others	7	9	16 (2.9)		3	8	11 (2.1)	
*Existence of assets that complement food source, specifically commercial farmland, buildings, machines, motor-vehicle, motor-cycle, bicycle, livestock or poultry*								
Yes	93	17	110 (19.8)	< 0.01	143	84	227 (43.4)	< 0.01
No	192	253	445 (80.2)		117	179	296 (56.6)	
*Received relief food in the last 3 years preceding the interview*								
Yes	65	27	92 (16.6)	< 0.01	242	4	246 (47.0)	< 0.01
No	220	243	463 (83.4)		18	259	277 (53.0)	

The level of education of the household heads differed significantly among the affected and controls (P < 0.01 in both districts), but generally more than 85% in both districts had not obtained an education beyond primary school. Affiliation to religious faith denominations varied among the affected and controls (P < 0.01 in both districts) with a majority affiliated to the Anglican faith; over 52% and 35% in the Bududa and Kiryandongo districts, respectively. Other differences between affected and controls in both districts were linked to ownership of assets that complemented food source such as land and other income generating assets, and the household having received relief food in the three years period preceding the interview. Although farming constituted the main source of livelihood in both the Bududa (90%) and Kiryandongo (86%) districts, a difference in the main source of livelihood between the affected and controls was observed in the Bududa district only (P < 0.01).

### Perceptions on the right to adequate food during disasters

#### Awareness of the right and the related state obligation to provide relief food

Varied responses were received on the structured questions about the right to adequate food and disaster relief food in Uganda (Table 2). The question on whether everyone had a right to adequate food received different responses among the affected and controls in both districts (P < 0.01 in both). Despite a majority in the Bududa (67%) and Kiryandongo (59%) answering in the affirmative, more than one in four respondents in both districts said that they did not know.

**Table 2:** **Perceptions on the right to adequate food and relief food during disaster in Uganda**

**Question**	**Bududa district (n = 555)**			**P values**	**Kiryandongo district (n = 523)**			**P values**
	**Affected (n = 285)**	**Controls (n = 270)**	**Total (%)**		**Affected (n = 260)**	**Controls (n = 263)**	**Total (%)**	
Do you agree that everyone has a right to adequate food?								
Yes	152	219	371 (66.8)	< 0.01	159	147	306 (58.5)	< 0.01
No	34	24	58 (10.5)		83	27	110 (21.0)	
Do not know	99	27	126 (22.7)		18	89	107 (20.5)	
Whether the 2010 disasters in Bududa affected access to adequate food								
Yes	277	257	534 (96.2)	0.15	257	155	412 (78.8)	< 0.01
No	4	11	15 (2.7)		0	83	83 (15.9)	
Do not know	3	2	5 (1.0)		3	18	21 (4.0)	
Whether satisfied with emergency response to the 2010 disaster in Bududa								
Yes	99	110	209 (37.7)	0.09	91	111	202 (38.6)	< 0.01
No	184	154	338 (60.9)		168	128	296 (56.6)	
Do not know	2	6	8 (1.4)		1	24	25 (4.8)	
Who is supposed to supply relief food during disasters?								
Government	218	225	443 (79.8)	0.04	240	214	454 (86.8)	< 0.01
NGOs	14	7	21 (3.8)		16	33	49 (9.4)	
Both	17	20	37 (6.7)		2	1	3 (1.0)	
Do not know	36	18	54 (9.7)		2	15	17 (3.3)	
Whether the relief food supplied to Ugandans affected by disaster is of good quality								
Yes	150	196	346 (62.3)	< 0.01	120	96	216 (41.3)	< 0.01
No	82	44	126 (22.7)		140	145	285 (54.5)	
Do not know	53	30	83 (15.0)		0	22	22 (4.2)	
Whether the relief food supplied in Uganda is sufficient in quantity								
Yes	83	39	122 (22.0)	< 0.01	43	43	86 (16.4)	< 0.01
No	150	211	361 (65.0)		211	194	405 (77.4)	
Do not know	52	20	72 (13.0)		6	26	32 (6.1)	
What could be the most preferred approach to ensure the right to adequate food of disaster victims								
Relief food	43	48	91 (16.4)	0.04	12	29	41 (7^.^8)	< 0^.^01
Cash	109	76	185 (33.3)		66	108	174 (33^.^3)	
Land for food production	133	146	279 (50.3)		182	126	308 (58^.^9)	

Whereas it is an established fact in human rights instruments that it is the obligation of the state as a primary duty bearer to provide adequate food to vulnerable citizens [3,18], we noted a difference in responses between affected and controls in both districts on the question of who is supposed to supply relief food during disasters (P = 0.04 in the Bududa district and P < 0.01 in the Kiryandongo district) (Table 2). Worth noting is that a combined total of 80% in the Bududa district and 87% in the Kiryandongo district said it was the Government that was supposed to provide relief food. There was also a significant contrast in response of the affected and controls on the question whether relief food supplied to Ugandans affected by disaster is of good quality (P < 0.01 in both districts). Whereas a total of 63% of the respondents in the Bududa district responded in affirmative, in the Kiryandongo district a majority 55% said it was not. There were different responses received from the affected and control households in both districts on the question whether relief food supplied in Uganda were sufficient in quantity (P < 0.01 in both districts). A total of 65% in the Bududa district and 77% in the Kiryandongo district said the quantity of relief food supplied in post-disaster situations was insufficient.

#### Inadequate dry rations of relief food and no opportunity for choice and preference

Unlike in the Bududa district, a difference in responses was noted in the Kiryandongo district between the affected and controls on the questions whether the 2010 landslide disaster could have affected access to adequate food (P < 0.01), and whether the disaster response to that disaster was perceived as satisfactory (P < 0.01). In both cases a majority of both affected and controls in the district said that disaster affected access to food (79%) but the Government response fell short of being satisfactory (57%).

In all the FGDs that were held, the participants’ opinions seemed unanimous on the issue of inadequacy of relief food. Indeed several participants said they knew and had witnessed that the main types of relief food supplied by the Government and other humanitarian agencies was mostly comprised of dry rations of maize corn (*Zea mays*) flour and legumes especially beans (*Phaseolus vulgaris*). Some different responses were noted among affected and controls in each district as follows.

#### Bududa district

Given that emergency relief and humanitarian assistance had been witnessed over a long period in the Bududa district in the aftermath of the 2010 landslide, participants in the affected and controls discussed this subject in fair detail.

Firstly, all discussants in the affected group declared that they had received relief food assistance at some point in time after the 2010 landslide had occurred but they were no longer receiving it at the time of the study. They said that the food mostly comprised dry maize corn and beans of varying quantities depending on availability, and they were given rations for a period of two weeks and in some cases a monthly ration. However, most participants said that the relief food they received was often not sufficient. One man said: *“What we receive is small but we have to live with it.”* On probing if there was any opportunity for choice and preferences during relief food distributions, several affected participants said this was not easy to achieve. One member said: *“That is expecting too much.”* Another referred to a famous proverb used in one of the local dialects called Luganda that literally says: *“You only complain about having received a small piece of meat if you have it firmly in your possession.”* As such, it seemed incomprehensible to demand more choices yet the amounts of what was being received were perceived as inadequate and more of a generous offer than an inherent right.

In the control group of Bududa district, some members said it had been possible for some people to access relief food even when they were not residents from the affected areas. Like their counterparts who were affected, the controls in Bududa district described that it was common that relief food supplied in Uganda was not enough. One woman said that: *“Assistance to disaster victims should be increased because the lives of these people have been completely destroyed.”* In another meeting, men described with empathy the lack of special consideration towards vulnerable children and elderly during the distribution of relief food. One of them said: *“Children and elderly need special consideration but you find them giving every family one ration … even elders’ line up for long hours to get their rations.”*

#### Kiryandongo district

Members of the affected community in the Kiryandongo district were critical about the lack of a variety of relief food supplied to them. One male member raised a rhetorical question by asking: *“What variety can you achieve when the only relief food you are given is dry beans and maize that are even not enough?”* Several other participants described how difficult and challenging it was to prepare dry food when one has been rendered destitute by a disaster. One affected women summed the mood by saying: *“The first struggle is to get the food ration but the other is how to cook it…I wish they could also give us firewood to use for cooking or cooked food.”* However, some affected participants in the Kiryandongo district mentioned that on isolated occasions, other items like cooking oil, sugar, salt and rice were provided by what they called *“government people”, “humanitarian agencies”,* and *“sympathisers.”* Despite our probes, participants could not confirm having witnessed relief food distributions that included other nutritionally relevant food varieties such as fruits, vegetables, bread, meat, fish, milk or ready-to-eat rations. Although they desired to have such varieties, many expressed pessimism if it would ever happen for a diversity of food options to be part of the disaster relief in the near future. One member seemed less hopeful and said: *“It is a dream that cannot happen.”*

In the control group of Kiryandongo district, participants in the FGDs were less enthusiastic when discussing relief food issues as compared to their affected counterparts after all many said they had not received any form of relief in many years. However, like all their counterparts, most discussants seemed to express a consistent view that relief food in Uganda was generally inadequate in both quality and quantity. In addition, the issue of responsiveness to disaster relief was expressed as a concern and linked to active leadership. A male participant linked the problem of slow dispatch of relief to weak leaders by saying: *“In some places and in some districts, relief food is delivered immediately, but if you have leaders who are not active it will take long.”* Another member, who was a member of one of the village executive complained that relief food was never forthcoming when Kiryandongo residents faced problems of disaster. He said: *“When lightening killed our children and floods affected our crops we never got any assistance from government.”* On choice and preference of relief, a member in the women discussant said: *“It was not easy to achieve.”* On probing this matter further, a participant retorted: *“You sell what you receive if you want choice and preference.”*

#### Land for food production is essential in addressing the right to adequate food

On the question regarding the most preferred means to ensure the right to adequate food of disaster victims given a choice of relief food, cash, or land for food production, there were significant differences in responses between the affected and control households in both the Bududa (P = 0.04) and the Kiryandongo district (P < 0.01). However, the affected and control households in both districts preferred the provision of land for food production as the outstanding choice to ensure the right to adequate food of disaster victims; over 50% of the interviewed households in the Bududa district and 59% in the Kiryandongo district preferred land as a preferred means to ensure the right to adequate food. Apparently, relief food was the least preferred means for ensuring the right to adequate food by both affected and controls households in the two districts.

Like with the structured response from the surveyed households, the FGD aspects on food and diet aroused sentiments about the land issue in the context of the widely practiced subsistence farming; mostly arguing that it was the main means for food production and livelihood. Although these issues were a prominent feature in most of the discussions, they took on different contexts among affected and control discussants in both districts.

#### Bududa district

In the control group of the Bududa district, the land issue was discussed prominently in the context of their being less available land for food production. A female participant linked the challenge to population growth: *“There is a population problem … land has been squeezed and soil fertility has reduced.”* The members of the control group in the district also mentioned of the failure to de-gazette some of the land protected by the State under the Mount Elgon Forest Reserves. To them, areas for food production were diminishing amidst growing population pressure, limiting food availability and food security in their areas which border to the Mount Elgon; considered by the Government authorities as a high risk zone for landslides. Another participant said: *“Government should open the boundaries of the forest for people to plant crops.”*

In the affected sub-county of the Bududa district, FGD participants complained that some of their land had been mapped and zoned-off as a landslide-disaster risk zone. They said community members had been denied access to their gardens on the basis of disaster-risk without viable alternatives. One man said: *“Some of our gardens have been fenced off and the government does not want us to farm in the mountain.”* Another sounded in despair that: *“We have little alternatives where to grow food these days.”* In addition, decline in soil fertility was also discussed as a problem by most participants. Some residents blamed landslides for poor yields in the Bududa district. One participant reckoned: *“We are living in hard times… the landslide was like a curse because these days soils are becoming barren and crop yields are low.”*

In relation to food and nutrition, poor diet was cited as a common problem for the community. Most participants in the affected and control groups linked this challenge of inadequate diet to lack of the means for accessing adequate food, mainly due to the necessitous problem of low incomes. Several participants described how they no longer had surplus food to sell for money to procure other household needs. Nearly all of the discussions painted an impression of absolute necessity in both the control and affected groups as hardly did the words *“money”* and *“poverty”* miss-out in these discussions. In one meeting held with households of the control group in Bududa district, a woman participant summed up the effect of inadequate means to access to food by saying: *“We now eat only one meal a day because there is no money to buy food…in some homes the children are surviving on maize porridge only”*. Similar opinions about challenges of not affording adequate food were common in the control groups in the district.

Among the affected group in Bududa, the problems associated with inadequate diets of less variety were also linked to low incomes in similar ways to the controls. One of the participants narrated how difficult and expensive it was to achieve a varied diet: *“If you are to eat more food varieties then you need more firewood and time to prepare…but even cooking with oil and salt are not easy to afford on a daily basis”*. In another group, a youth described that competing needs often required that fruits and vegetables harvested by the household are sold for money rather than eaten: *“We cannot eat many foods like that yet we have many needs like school fees, clothing…when fruits and vegetables are in season, we harvest and sell them to meet other needs.”* There was an impression that a variety of food in the diet was dependant on incomes and wealth levels. In effect, there was a continuous tendency for discussions in both districts to skew towards poverty and low incomes whenever diet related issues were being discussed.

#### Kiryandongo district

In the discussions about the response to the 2010 landslide, the issue of resettlement of some of the victims into the Kiryandongo district was a dominant topic, especially issues of the ownership rights on the resettlement land and how it related to exercising the right to adequate food. However, sharp contrasts based on tribal and ethnic backgrounds were observed in this district. On one hand, the affected group comprised landslide victims who were a Lumusaba speaking community resettled from the Bududa district located on the foot of Mount Elgon in Eastern Uganda and therefore accustomed to the Bamasaba culture and values. On the other hand, the control group comprised mainly indigenous natives who were a Runyakitara speaking community accustomed to the culture and values associated with the Bunyoro-Kitara kingdom and heritage of Western Uganda. The apparent diversity in culture, ancestral heritage and circumstances between the two groups was also expressed in their opinions during the FGDs.

In the affected group, although most discussants hailed the Government programme of resettling them into the Kiryandongo district, many also argued that more needed to be done in respect to the 2010 landslide disaster response. Some said it was difficult to resettle in a community where the culture and traditions were different from where they came from. One man asked a question rooted in traditional culture: *“What about our ancestors? …can you imagine going to live somewhere you do not speak the language and have no relatives?”* Another added: *“The best solution is to move people nearby to their ancestral home and district…we have plenty of empty land and forests around Bududa district.”* In effect, the affected discussants wished they could gain ownership of the land onto which they had been resettled. A participant expressed pessimism by saying: *“Life is hard and our future here is not clear …we are not sure whether this land belongs to us.”* Another added: *“Some of our colleagues are returning to Bududa because this land is not ours …we wish they can give us permanent ownership.”*

The controls in the Kiryandongo district seemed unhappy when they discussed the Government resettlement of disaster affected households and other people into the district. In all the three meetings that were separately held with women, men and the youth, participants complained, in some cases bitterly, that people from other districts were being resettled in their district despite many of their own folk being landless and living in deprivation. One dominant male participant received remarkable applause from his colleagues when he said: *“Kiryandongo is now like a dumping ground for disaster victims and refugees…we no longer have grazing land and food is being stolen by some of these people.”* Another questioned: *“Who was consulted for them to come here?”* The discontent and feeling of resentment expressed by the participants in the control groups of the Kiryandongo district indicated that the resettlement and integration process of the disaster affected victims was still a matter that leaves a lot to be desired. One could observe that either the local community members might not have been consulted about the resettlement or they were not comfortable with the reality that the Government had resettled landslide victims into their neighbourhood.

### Perceptions about human rights principles of participation, non-discrimination, accountability and transparency

The discussions on the topic about human rights-based approach were entirely captured from the FGD. We generally noted that the discussions were shallow and left a lot to be desired. We consistently observed that the reasons behind the poor discussions were linked to a low awareness and comprehension across all groups of participants among the affected and controls in both districts. Suffice to mention that most participants interpreted the topics about participation in decision making, non-discrimination in relation to relief food during disaster management, and accountability and transparency as being challenging to them. As such, a few participants attempted to discuss them while many others often kept silent despite an effort to encourage them to speak openly and freely. In effect we present here key excerpts from the general issues that were noted in the two districts combined given that differences could not be clearly deduced from the few responses of the affected and control participants.

Most participants contextualised the issue of participation in the decision making process as being exercised through representation by their leaders. One male member from the affected group in Bududa district said that: *“The chairman is always around to meet with those people from the government when we have problems.”* Similar views expressing how the local leaders represented their views in decision making were echoed in all groups in both districts. Other participants, especially in the Bududa district, likened participation with representation by elected leaders including Members of Parliament (MPs). Accordingly, one female participant drawn from the controls said: *“We rely on our MP for information about impending government support.”* Participants also described that committees involving their leaders were usually active when disasters happened, and that those leaders acted as their representatives on issues of participation when dealing with the Government and relief agencies. However, it also seemed apparent that the continuous reference to the phrase *“chairman”* and not chairwoman or chairperson, gave the impression of a possible male dominated leadership in the two districts.

The issue of non-discrimination in the context of access to relief food was mostly discussed with caution and in some cases it nearly aroused hostile political debate. Some discussants on this subject referred to examples incriminating local politicians and leaders despite a caution to avoid naming and victimization. In the Bududa district, some affected male participants said they were privy to scenarios when some leaders engaged in tendencies to side-line some people from benefiting from some of the relief operations. Accordingly, some district leaders and politicians, often referred to as *“top officials”* were influential during food mobilisation and distributions. Interestingly, one female participant said: *“Some leaders will care less when disasters affect where they did not get many votes.”* Another affected participant who claimed to have missed out on the resettlement programme said: *“I was not included on the list to go to Kiryandongo because the leader who was involved does not like me.”*

The issues of accountability and transparency were discussed with the least interest in both districts. Their interpretation also received mixed impressions. Accountability was mostly narrowed to knowledge of how much financial and material resources are being used in disaster response operations and whether or not these resources are put to their intended use. On the other hand, transparency was mostly narrowed to imply the easy availability and access to all the information about the activities and resources being used. One male participant in the control of Kiryandongo district said: *“Those are complex political matters.”* Although most participants could not contribute to the discussions on these matters by saying they did not know much, a participant in the Bududa district equivocally stated that: “*Those matters are decided in Kampala* (Uganda’s capital city).*”* Another expressed that accountability was a preserve of those who had attained some education by retorting that: *“How can we know about accountability when many of us are not educated like you people?”*

In the last FGDs held with the control groups at the Kiryandongo sub-county headquarters, participants described the failure in the transparency processes within the context of lack of honesty and trustworthy actions. One euphoric discussant was quick to assert that: *“It is not easy to be transparent on matters of relief food…you cannot trust people to be honest when they look at the suffering of others as an opportunity to gain wealth.”* Similar sentiments about possible mistrust of the authorities were commonly said.

Though most of the discussions in both districts painted an impression of generally low levels of awareness of human rights principles, the challenge was more pronounced with the principles of accountability and transparency; the issues seemed to be challenging to discuss and were not comprehensively explored despite persistent probes. It also seemed apparent that the whole subject of a rights based approach and human rights principles pose contextual challenges for rights holders to easily and fully comprehend the substance matter of the various principles and normative standards (rights).

## Discussion

The main findings showed that there were significant differences in household characteristics between affected and controls in both districts, especially with regard to the head of the households’ age, education level, religious affiliation, ownership of relevant assets that complement food source, having benefited from relief food and the main source of livelihood. Apparently, rights holders from affected and control households in the Kiryandongo district also provided significantly different responses to all the seven structured questions about the right to adequate food and relief food in Uganda: whether everyone has a right to adequate food; whether the 2010 landslide disaster in the Bududa district affected access to adequate food; whether the emergency response to the 2010 disaster was considered satisfactory; who is supposed to supply relief food in Uganda; whether relief food supplied to Ugandans is of good quality; whether relief food supplied to Ugandans is of sufficient quantity; and the preferred means to ensure the right to adequate food of disaster victims. In the Bududa district the differences were noted on five questions, with exception of two questions on whether the 2010 landslide disaster affected access to adequate food, and whether the emergency response to the 2010 disaster was considered satisfactory (Table 2).

Moreover, a majority of rights holders in both districts answered in the affirmative, and therefore seemed aware, that everyone has a right to adequate food, and that the provision of relief food was the responsibility of the Government. However, the quality and quantity of relief food supplies in Uganda were cited as often being inadequate by most respondents, while availability of land for food production was the most preferred intervention for ensuring the right to adequate food in the aftermath of a landslide disaster, albeit the challenge of diminishing land for food production.

Outcomes from the FGD also pointed to some differences in perceptions between the affected and controls in each district. In the Bududa district, the affected group highlighted that mapping most of their land as being part of the disaster-risk area had negatively affected food production, and the landslides had deprived them of land, incomes and property, which are vital means for one to have adequate food. The relief food they received was limited in quality and quantity with no choice and ability to pursue their own preference. Control participants in the district pointed to the problem of diminishing land that was leading to deficits in food production and income levels, restrictions from using gazetted forests as a food source, and low incomes to afford a varied diet. In the Kiryandongo district, the affected people were pessimistic about their ownership rights of the land onto which they were resettled and expressed concern that they had no opportunity to make choice when receiving relief food. On the other hand, the control group was unhappy and concerned about the absence of a clear national policy and standard procedures to resettle disaster victims in their district when their own folk did not have land. They expressed dissatisfaction that the Government approach to relief and resettlement did not offer opportunities for consultations and concessions. Despite an attempt to discuss human rights principles of participation and non-discrimination in the context of disaster and relief food, comprehension was low and there were was general apathy towards discussing issues of accountability and transparency. The latter principles were perceived as complicated subject matters for rights holders to easily comprehend and discus. Moreover, education levels were low with a majority having not exceeded primary school.

These findings unveil a mismatch of realities and expectations. On the one hand, the rights holders seem to be aware, albeit in less detail, that they have a right to adequate food, and that the State has the primary obligation to provide disaster relief food. However, despite admission that relief food is often inadequate, people did not trust that it is the most desired means to achieve their right to adequate food; land for food production was still the widely preferred choice as the means to realise the right to adequate food of disaster victims. In addition to challenging the sustainability of relief food, these perceptions also seem to concur with the arguments of Sen [29], Dreze [30] and Mechlem [31] that the right to adequate food is too complex to be delinked from other complementary economic and social rights and entitlements. This case also reinforces the Vienna declaration stating that: *“All human rights are universal, indivisible, interdependent and interrelated”* [32]. In effect, though hunger and starvation prevention should be an urgent priority during disaster situations, it should not be the only and ultimate priority in interpreting what constitutes the interventions to realise the right to adequate food. A broader human rights approach that addresses a host of supportive amenities related to the right to adequate food (e.g. people’s contribution through participation, accountability in the management of the relief operations, and openness and transparency in the process) is of essence in such situations.

Despite the role played by humanitarian agencies, especially the Uganda Red Cross which appears to be very active in the supply of disaster relief food during the landslides and floods that were subsequent to it [33], the perception that the state should to do more with regard to assurances on the adequacy of relief food and guarantees for land ownership during resettlement, serves to add emphasis to the state obligations of conduct on the right to adequate food. According to Eide [34], these obligations of conduct require the government to undertake purposeful sought out and calculated actions to enable the population, especially the most vulnerable, to realize and exercise their right to adequate food. Hence, to demonstrate action requires that responsive policy and legal measures are adopted so as to institute mechanisms that assure each citizen’s access to adequate food at all times.

Furthermore, significant resources can be saved if the state avails an array of short and long-term measures grounded in implementable policy, legal and institutional arrangements to mitigate hunger and assure nutrition security, in the wider context of ensuring an adequate standard of living for disaster victims [35]. Moreover, tools on the right to adequate food have been adopted by United Nations [3,4]. These tools, if domesticated, would be helpful in complementing existing programmes since they provide an elaborate mechanism state and other actors can progressively realise this right, even in circumstances of disaster and within the general context of national food security.

These findings from rights holders’ seem to correspond with observations from another study on Uganda’s duty bearers [36]. Both studies have shown that the right to adequate food during disaster preparedness and emergency response in Uganda is complex in terms of implementation. There is a problem of capacity constraints faced by the relevant sectors responsible for food and nutrition security [13,37,38] and disaster management [36]. As such, non-state humanitarian actors including the Uganda Red Cross seem to have a noticeable presence at the operational level. Though humanitarian agencies provide a complementary role to the state, entrenching their approach that is often bound by humanitarian law may be contradictory to the state obligation on human rights, which place the primary obligation on the state. As much as the “save life” approach used by humanitarian actors may be preferred as an immediate economically cost-reducing option to ensure the right to be free from hunger and extreme outcomes of starvation, its entrenchment often suppresses human dignity by not taking into account adequacy, choice and preference [39,40]. Moreover, there is often no demonstrated proof that the state has taken steps to the maximum of their available resources in order to provide adequate relief as part of the efforts to realise the right to adequate food. Failure by the state to demonstrate this commitment in time of disaster relief operations may in effect constitute a breach of Article 2(1) of the International Covenant on Economic, Social and Cultural Rights [2].

The complexity of assuring the right to adequate food during disaster situations is also rooted in the design and governance approaches in the international humanitarian and human rights regimes. In spite of the mutual connectedness of the doctrines of humanitarian law and human rights law, adequacy of relief food as a human right is often not rigorously interpreted by the state and the non-state actors in the form and substance specified by international human rights instruments [39-41]. Besides, a United Nations legislative study by Cotula and Vidar [35] has described how the standards often are incompatible but need to be harmonised. They argued that the supreme approach of the state, as the lead agency, should not be restricted to a minimalist humanitarian approach that is viewed in the economic sense of saving money, but rather a holistic domestication and implementation of a right to adequate food-approach in all situations, including during natural and human induced disasters.

In spite of the merits for utilising both qualitative and quantitative methods in this study, a number of limitations should be taken into consideration when interpreting the findings. The cross-sectional nature of the design restricts the possibilities of drawing causal inference [42], while some of the perceptions could have also been influenced by the different ecological and socio-demographic characteristics and conditions that prevailed at the point in time of the study. Additionally, whereas the qualitative approach involving focus group discussions is described as a labour intensive technique [43,44], it was suitable and of added value in facilitating the process of exploring the rights holders’ perceptions to a level beyond what could be gained through quantitative methods alone. However, although we segmented focus group participants into youth, adult men and women, and further notified them that all of their opinions would be considered without bias so that they could discuss freely and without fear, it is possible that some participants may not have freely expressed their opinions due to the attendance of others who might have been acquaintances or adversaries [45,46]. Moreover, perceptions held by affected households on relief food and coping experiences may have been skewed in favour of their needy situation, a phenomenon that Maxwell and Caldwell [47] refer to as the learning effect: a common inaccuracy when respondents report more or less than actually happens to them in order to ensure that their household remain as a potential beneficiary.

## Conclusion

It is apparent from this study that the right to adequate food in situations of disaster and emergency response is complex. The findings point to a number of issues that rights holders’ wanted addressed as a means of improving their availability and access to adequate food in the aftermath of landslides. Accordingly, the perceived standards and expectations during critical circumstances of disaster, the inadequacy of disaster relief food and the failure to meet anticipated remedies and recourse after a disaster, were often not taken into consideration. Although the perceptions of rights holders about the right to adequate food might have been influenced by experiences related to specific events that affect this right, in this case landslides, their views seem to question whether a human rights-based approach to disaster management had gained desirable ground in the country.

Moreover, whereas in this study the perceptions on relevant human rights principles such as participation and non-discrimination can be considered as rather promising, the debate on accountability and transparency among rural folks seemed to be weak. Although this could arise when education levels are low as witnessed in this study, it also points to a possible failure in translating relevant human rights tools to communities in the country. It is also apparent that relevant information and awareness promotion platforms targeting rights holders’ on issues of relief food accountability and transparency are lacking. This seems to have bred perceptions that such information is a preserve of members in the top echelons of society, especially the educated elites and persons in higher positions of authority.

Going forward, the rights holders’ perceptions paint an impression that those consulted aspire to enjoy the inherent liberties and entitlements associated with this right especially land for own food production; implying, they prefer facilitation of own food production rather than provision of food rations. To support their bid, a robust resettlement policy may be of the essence to address the land issue in post-landslide displacement and deprivation. Additionally, relevant tools and information materials about human rights awareness need to be assembled and made available to empower the citizenry, especially in rural areas, on their right to adequate food. Such an investment in capacity building may boost public awareness and perceptions and enhance demand for desirable and concrete actions. That approach may also build the potential to improve the rights holders’ full participation in the monitoring of accountability and transparency in food and nutrition programmes. In effect, remedy and recourse mechanisms in the aftermath of disasters need to be clearly defined in legislation, if duty bearers are to be held accountable for their actions, which by omission or commission, may interfere with the right to adequate food during disasters.

## Abbreviations

FGD: Focus group discussions
GC 12: United Nations General Comment Number 12 on the right to adequate food
VGs: Voluntary Guidelines to support the progressive realization of the right to adequate food in the context of national food security
